# Patients with first recurrent retroperitoneal sarcoma that can be macroscopically completely resected can achieve comparable outcomes with that of primary patients after *en bloc* resection of tumor and adjacent organs

**DOI:** 10.3389/fsurg.2022.956384

**Published:** 2022-09-07

**Authors:** Zhen Wang, Jian-hui Wu, Cheng-peng Li, Ang Lv, Hui Qiu, Xiu-yun Tian, Bo-nan Liu, Chun-yi Hao

**Affiliations:** Key Laboratory of Carcinogenesis and Translational Research (Ministry of Education, Beijing), Department of Hepato-Pancreato-Biliary Surgery, Sarcoma Center, Peking University Cancer Hospital and Institute, Beijing, China

**Keywords:** retroperitoneal sarcoma, recurrence, *en bloc* resection, macroscopically completely resected, retroperitoneum

## Abstract

The outcomes of patients with primary retroperitoneal sarcoma (RPS) are significantly superior to those with recurrence. *En bloc* resection of tumor and adjacent organs is recommended in primary RPS. However, whether *en bloc* resection of tumor and adjacent organs can benefit recurrent patients or some recurrent patients is unclear. We compared the outcomes of patients with primary RPS, first recurrence (RPS-Rec1), and ≥2 recurrences (≥RPS-Rec2) to evaluate the value and criteria for *en bloc* resection of tumor and adjacent organs in recurrent cases. We evaluated the safety of *en bloc* resection of tumor and adjacent organs by assessing operation time, blood loss volume, postoperative morbidities (POM), and efficacy by comparing local recurrence and peritoneal metastasis (LR-PM), distant metastasis, progression-free survival (PFS), and overall survival (OS). A total of 101, 47, and 30 patients with primary RPS, RPS-Rec1, and ≥RPS-Rec2 were included, respectively. Recurrent RPS invaded more adjacent organs and surrounding fat tissue than primary RPS. The operation time, amount of blood loss, incidence of grade III–V POM, LR-PM rate, PFS, and OS in the RPS-Rec1 group were similar to those of the primary group, both of which were significantly superior to those of the ≥RPS-Rec2 group. Macroscopically incomplete resection and high-grade RPS rather than first recurrence were independent risk factors for LR-PM, PFS, and OS. In conclusion, the safety and efficacy of *en bloc* resection of tumor and adjacent organs in RPS-Rec1 were comparable with those in primary RPS but significantly superior to those of ≥RPS-Rec2. For RPS-Rec1, comparable outcomes to patients with primary RPS can be achieved, particularly in those in whom a macroscopically complete resection is achieved.

## Introduction

Approximately 15% of soft tissue sarcomas are located in the retroperitoneum. Of all pathological types, liposarcoma is the commonest (1, 2). Surgical resection is the mainstay treatment for retroperitoneal sarcoma (RPS). Local recurrence is common after first resection, with a five-year local recurrence rate of approximately 50% (2–7). Local recurrence is the leading cause of death in patients with RPS, with up to 70% of deaths occurring in the absence of distant metastasis (3, 8, 9). To reduce local recurrence after primary resection, achieving complete resection by *en bloc* resection of tumor and adjacent organs is recommended (4, 5, 10, 11).

The outcomes of patients with primary RPS is significantly superior to that of recurrence, and the complete resection rate significantly decreases with each subsequent local recurrence (3, 12). Based on the above research results, some scholars recommend a relatively conservative approach for treatment of locally recurrent RPS as opposed to a more liberal approach for the primary tumor as the probability of curing the patient is low (13). Several studies have demonstrated that patients undergoing surgery for first locally recurrent retroperitoneal sarcoma (RPS-Rec1) have better survival than those who do not undergo the surgery (14). However, these studies did not clarify which patients can benefit from surgery and which patients should not receive surgical treatment. Thus far, it is as yet unclear whether *en bloc* resection of tumor and adjacent organs involvement is advisable for patients with certain recurrent RPS.

By comparing patients with a first local recurrence of RPS, primary RPS, and ≥2 local recurrences (≥RPS-Rec2), this study aimed to evaluate the value of *en bloc* resection of tumor and adjacent organs and select cases in recurrent RPS patients.

## Material and methods

### Patients

We retrospectively analyzed patients with primary and locally recurrent RPS at Peking University Cancer Hospital Sarcoma Center (PUCHSC) between March 2009 and October 2019. Our surgical policy is to perform extended *en bloc* resection of the tumor along with adjacent organs. All the patients included in this study underwent surgery with a curative intent. Liberal *en bloc* resection of surrounding tissues and organs was conducted when they were located within 1 to 2 cm from the tumor surface. Pancreaticoduodenectomy was conducted when the tumor infiltrated the pancreatic head and greater part of the duodenum. If necessary, great vessels (such as the inferior vena cava, aorta and iliac vessels) were removed and replaced with polytetrafluoroethylene grafts. If the diaphragm and/or pericardium showed tumor invasion, partial diaphragm and/or pericardium resection and repair were performed (15). All patients gave informed consent according to the procedures required by the Institutional Review Board of Peking University Cancer Hospital and Institute and in accordance with the Declaration of Helsinki.

### Pathological diagnosis

All tumors were delivered to the pathology receiving room after the operation. Overall tumor size was defined as the sum of the maximum diameters of all the tumors at the time of the first surgical resection. The specimen were orientated by the surgeon, and all margins were perpendicularly sampled, with two or more sections taken from all margins. Additional sections were taken from the closest margin. Serial sampling of all resected organs and the surrounding fat was performed, and the tissues between the tumor and an organ were sampled every 2 cm. Two sarcoma pathologists independently confirmed the pathological diagnosis. The three-tier Federation Nationale des Centres de Lutte Contre le Cancer (FNCLCC) criteria was used for tumor grading (16).

### Definitions

The surgical policy was to remove the tumor with adjacent organs *en bloc*. Pancreaticoduodenectomy was conducted when the tumor invaded the pancreatic head and greater part of the duodenum. If necessary, great vessels (such as the inferior vena cava, aorta and iliac vessels) were removed and replaced with polytetrafluoroethylene grafts. If the diaphragm and/or pericardium showed tumor invasion, partial diaphragm and/or pericardium resection and repair were performed.

Surrounding fat was defined as the fat tissue within 1 mm from the organ surface. The invasion pattern was classified as organ parenchyma invasion (OPI) and surrounding fat invasion (SFI). OPI was defined as invasion of the bowel or parenchyma of solid organs. SFI was defined as invasion of the surrounding fat tissue only, without OPI. Both SFI and OPI were considered as organ invasions of RPS.

Since it was difficult to distinguish whether the recurrent retroperitoneal, abdominal, and pelvic tumors after surgery are local recurrence or peritoneal metastasis, we defined all the recurrent lesions located in retroperitoneum, peritoneal cavity, and pelvis as “local recurrence and/or peritoneal metastasis (LR-PM)”. “RPS-Rec1” was defined as LR-PM after the first resection surgery. “≥RPS-Rec2” was defined as ≥2 LR-PM after the resection surgery. *“*Distant metastasis” was defined as emerging lesions located in sites other than the retroperitoneum, peritoneal cavity, and pelvis, such as the lung, spine, etc.

“First surgery” was defined as curative-intent surgery for primary RPS. “Second surgery” was defined as curative-intent surgery for patients with RPS-Rec1. “Multiple surgery” was defined as curative-intent surgery for patients with RPS-Rec2.

The resection was classified as macroscopically complete resection (MCR) (R0 or R1) or macroscopically incomplete resection (MIR) (R2) based on the surgeons’ evaluation during the operation. Intraoperative tumor rupture was classified as MIR (17).

### Postoperative morbidities and follow-up

Postoperative morbidities (POM) were graded according to the seven grades of the Clavien-Dindo classification (I, II, IIIa, IIIb, IVa, IVb, and V) (18). The patients were prospectively followed with clinical examination, chest x-ray, and abdominopelvic computed tomography (CT) or magnetic resonance imaging (MRI) every three months for the ﬁrst two years, every six months for the next three years, and yearly thereafter.

### Data analysis

Data are presented as median and range, or number and percentage, where appropriate. Demographic and clinical characteristics were compared using the Pearson chi-Square and Fisher’s exact tests for dichotomous variables and the Mann–Whitney U test for continuous variables. Competing risk analyses were used to calculate the cumulative incidence of LR-PM and distant metastasis; the Gray test was used to compare between the groups (19). For all the patients, the local progression-free survival (PFS) and overall survival (OS) were analyzed. For patients who underwent MIR, PFS was defined as survival without tumor enlargement, new occurred tumor, or distant metastasis. For patients with residual tumor during operation, the time of LR-PM was the first follow-up review after surgery (one month). Survival probabilities were estimated using the Kaplan–Meier method. Potential prognostic factors for POM were evaluated by multivariate analysis using logistic regression. Potential prognostic factors for LR-RM, PFS, and OS after the operation were evaluated by multivariate analysis using Cox proportional hazards regression. Variables with a *P*-value ≤0.1 were considered in the multivariate models. Statistical analyses were performed using SPSS version 24.0 (Armonk, NY; IBM Corp) and R version 3.4.0 (http://www.r-project.org). *P*-values less than 0.05 were considered statistically significant.

## Results

### Patient characteristics

From March 2009 to October 2019, 192 patients with RPS underwent *en bloc* resection with adjacent organ involvement at our center. Three patients had distant metastases before the operation, one patient had concurrent lymphoma, and one patient had RPS metastases from other sites. Nine patients with missing follow-up data were excluded. A total of 178 patients were, thus, included in this study. There were 101 patients who underwent the first surgery, 47 had second surgery, and 30 had multiple surgeries. For those with recurrence, the previous operation was to remove the tumor alone, or only to remove the adjacent organs directly involved with the tumor. Ten patients received two times operations in our center (including 4 cases of primary + RPS-Rec1, 1 case of primary + ≥RPS-Rec2, 3 cases of RPS-Rec1 + ≥RPS-Rec2, and 2 cases of both operations are ≥RPS-Rec2). The other patients underwent only one operation in our center. The clinical characteristics of the patients are shown in Table 1. There were no significant differences in age (*P* = 0.311), sex (*P* = 0.082), pathological diagnosis (*P* = 0.421), and tumor grade (*P* = 0.117) between the primary, RPS-Rec1, and ≥RPS-Rec2 groups.

**Table 1:** Clinical characteristics of the patients.

Characteristics	Primary (*n* = 101)	RPS–Rec1 (*n* = 47)	≥RPS–Rec2 (*n* = 30)
Age (continuous variable)	57 (30–83)	56 (17–74)	57 (18–77)
Sex			
Male	57 (56.4%)	25 (53.2%)	10 (33.3%)
Female	44 (43.6%)	22 (46.8%)	20 (66.7%)
Perioperative therapy			
Yes	2 (2%)	6 (12.8%)	18 (60%)
No	99 (98%)	41 (87.2%)	12 (40%)
Diagnosis			
WDLPS	19 (18.8%)	3 (6.4%)	3 (10%)
DDLPS	30 (29.7%)	19 (40.4%)	17 (56.7%)
Myxoid liposarcoma	7 (6.9%)	3 (6.4%)	3 (10%)
Mixed-type liposarcoma	5 (5%)	2 (4.3%)	2 (6.7%)
UPS	8 (7.9%)	4 (8.5%)	0 (0%)
Leiomyosarcoma	19 (18.8%)	7 (14.9%)	3 (10%)
Others	13 (12.9%)	19.1 (8.5%)	2 (6.7%)
Tumor size	19 (4–66)	17.3 (2.3–92)	31.5 (4–62)
Tumor number			
Single	87 (86.1%)	22 (46.8%)	10 (33.3%)
Multiple	14 (13.9%)	25 (53.2%)	20 (66.7%)
FNCLCC grade			
1	24 (23.8%)	6 (12.8%)	2 (6.7%)
2	35 (34.7%)	17 (36.2%)	9 (30%)
3	42 (41.6%)	24 (51.1%)	19 (63.3%)

DDLPS, dedifferentiated liposarcoma; FNCLCC, Federation Nationale des Centres de Lutte Contre le Cancer; UPS, undifferentiated pleomorphic sarcoma; WDLPS, well-differentiated liposarcoma; RPS, retroperitoneal sarcoma; RPS-Rec1, first recurrence of RPS; ≥RPS-Rec2, ≥2 recurrences.

### Overview of the operation

**Figure 1:**
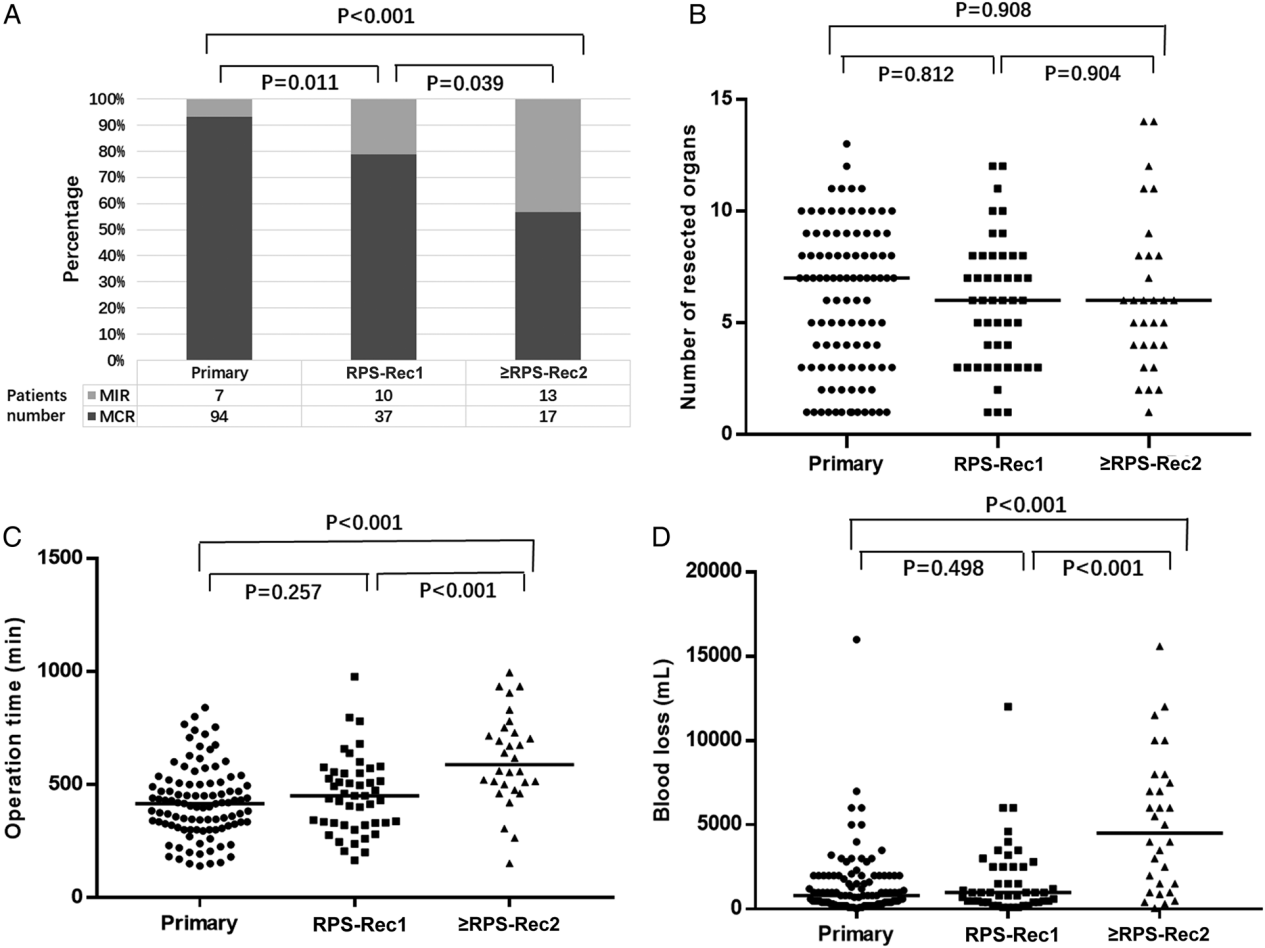
Overview of the operation. (**A**) Percentage of patients achieved MCR (primary vs. RPS-Rec1, *P* = 0.011; primary vs. ≥RPS-Rec2, *P* < 0.001). (**B**) Number of resected organs (primary vs. RPS-Rec1, *P* = 0.812; primary vs. ≥RPS-Rec2, *P* = 0.908). (**C**) Operation time (primary vs. RPS-Rec1, *P* = 0.257; primary vs. ≥RPS-Rec2, *P* < 0.001). (**D**) Blood loss (primary vs. RPS-Rec1, *P* = 0.498; primary vs. ≥RPS-Rec2, *P* < 0.001). Black lines indicate median values. MCR, macroscopically complete resection; MIR, macroscopically incomplete resection.

Ninety-four (93.1%), 37 (78.7%), and 17 (56.7%) patients with primary, RPS-Rec1, and ≥RPS-Rec2, respectively, achieved MCR (primary vs. RPS-Rec1, *P* = 0.011; primary vs. ≥RPS-Rec2, *P* < 0.001) (Figure 1A). The reasons for the failure of MCR are shown in Supplementary Table S1. The median number of organs resected in the primary, RPS-Rec1, and ≥RPS-Rec2 groups was 7 (1–13), 6 (1–12), and 6 (1–14), respectively (primary vs. RPS-Rec1, *P* = 0.812; primary vs. ≥RPS-Rec2, *P* = 0.908) (Figure 1B). Details of the organs resected in each group are shown in Supplementary Table S2. The median operation time was 415 (140–840) min, 450 (165–977) min, and 588.5 (152–995) min, respectively, in the primary, RPS-Rec1, and ≥RPS-Rec2 groups (primary vs. RPS-Rec1, *P* = 0.257; primary vs. ≥RPS-Rec2, *P* < 0.001) (Figure 1C). The median blood loss volume in the primary, RPS-Rec1, and ≥RPS-Rec2 groups was 800 (50–16,000) ml, 1000 (100–12,000) ml, and 4,500 (50–15,600) ml, respectively (primary vs. RPS-Rec1, *P* = 0.498; primary vs. ≥RPS-Rec2, *P* < 0.001) (Figure 1D).

### Overview of the invasion

**Figure 2:**
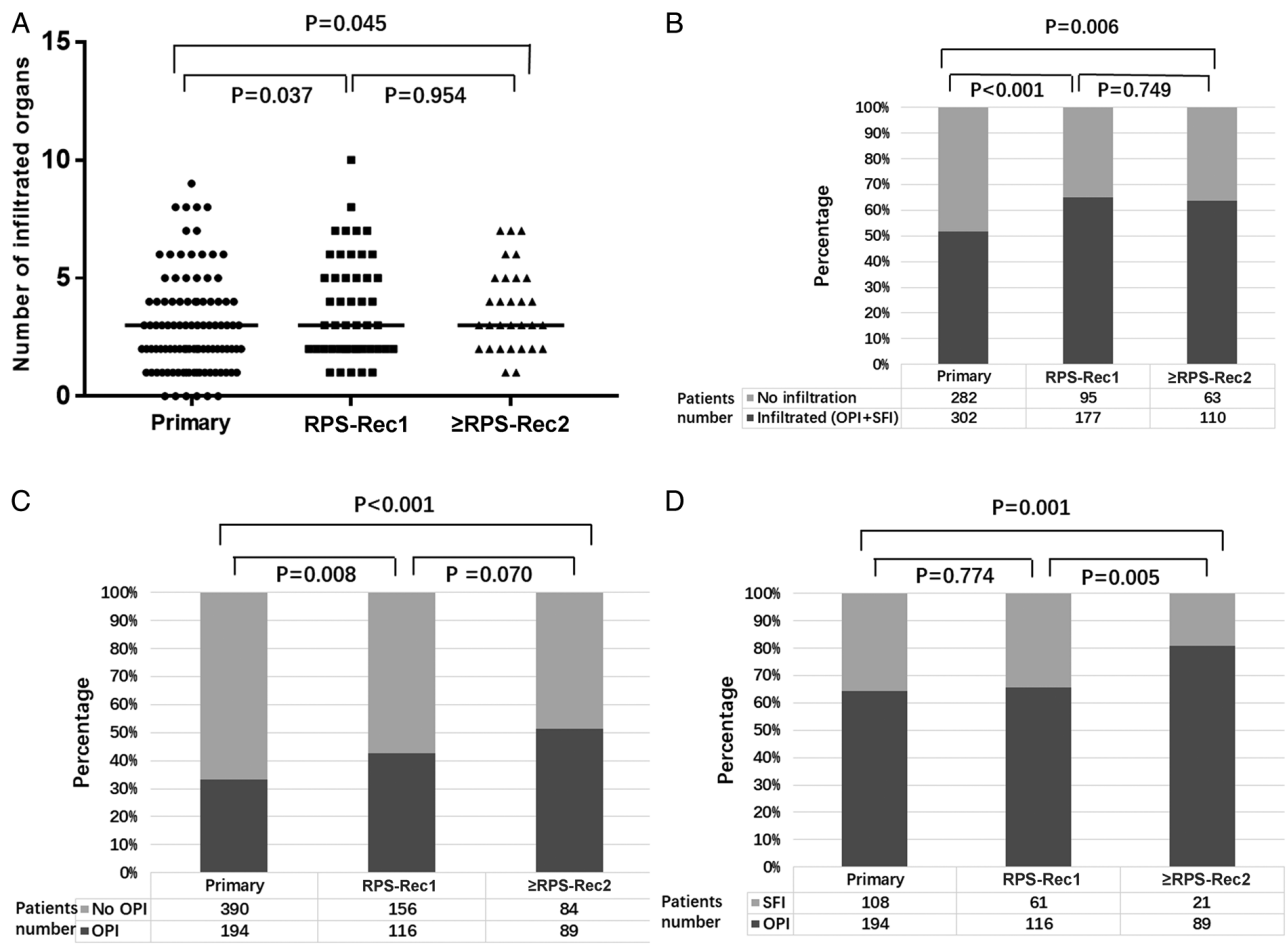
Overview of the invasion. (**A**) Number of invaded organs (including OPI and SFI) (primary vs. RPS-Rec1, *P* = 0.037; primary vs. ≥RPS-Rec2, *P* = 0.045). (**B**) Percentage of invaded organs (including OPI + SFI) (primary vs. RPS-Rec1, *P* < 0.001; primary vs. ≥RPS-Rec2, *P* = 0.006). (**C**) Percentage of OPI in all the resected organs (primary vs. RPS-Rec1, *P* = 0.008; primary vs. ≥RPS-Rec2, *P* < 0.001). (**D**) Percentage of OPI in the invaded organs (including OPI and SFI) (primary vs. RPS-Rec1, *P* = 0.774; primary vs. ≥RPS-Rec2, *P* = 0.001). Black lines indicate median values. OPI, organ parenchyma invasion; SFI, surrounding fat invasion.

The median number of invaded organs was 3 (0–9), 3 (1–10), and 3 (1–7) in the primary, RPS-Rec1, and ≥RPS-Rec2 groups, respectively (primary vs. RPS-Rec1, *P* = 0.037; primary vs. ≥RPS-Rec2, *P* = 0.045) (Figure 2A), and the percentage of invaded organs was 51.7% (302/584), 65.1% (177/272), and 63.6% (110/173), respectively (primary vs. RPS-Rec1, *P* < 0.001; primary vs. ≥RPS-Rec2, *P* = 0.006) (Figure 2B). Among the patients with primary, RPS-Rec1, and ≥RPS-Rec2, 33.2% (194/584), 42.6% (116/272), and 51.4% (89/173) of the parenchyma of the total resected organs was invaded by RPS, respectively (primary vs. RPS-Rec1, *P* = 0.008; primary vs. ≥RPS-Rec2, *P* < 0.001) (Figure 2C); 64.2% (194/302), 65.5% (116/177), and 80.9% (89/110) of the parenchyma of the invaded organs (including OPI and SFI) was invaded by RPS, respectively (primary vs. RPS-Rec1, *P* = 0.774; primary vs. ≥RPS-Rec2, *P* = 0.001) (Figure 2D).

### Postoperative morbidity

In the primary, RPS-Rec1, and ≥RPS-Rec2 groups, grade III–V POM occurred in 17 (16.8%), 13 (27.7%), and 12 (40%) patients, respectively (primary vs. RPS-Rec1, *P* = 0.127; primary vs. ≥RPS-Rec2, *P* = 0.007). Details of the POM of the first RPS and recurrent groups are shown in Supplementary Table S3. The multivariate analyses showed that great vessel resection (relative risk [RR]: 1.01; 95% confidence interval [CI]: 1.00–1.01; *P* = 0.017) and pancreatoduodenectomy (RR: 3.07; 95% CI: 1.02–9.26; *P* = 0.047) were independent risk factors for grade III–V POM. In the primary, RPS-Rec1, and ≥RPS-Rec2 groups, 8 (7.9%), 5 (10.6%), and 6 (20%) patients underwent reoperation due to POM, respectively (primary vs. RPS-Rec1, *P* = 0.817; primary vs. ≥RPS-Rec2, *P* = 0.123). The perioperative mortality of the primary, RPS-Rec1, and ≥RPS-Rec2 groups was 2%, 4.3%, and 10%, respectively (primary vs. RPS-Rec1, *P* = 0.592; primary vs. ≥RPS-Rec2, *P* = 0.063).

### LR-PM and distant metastasis

**Figure 3:**
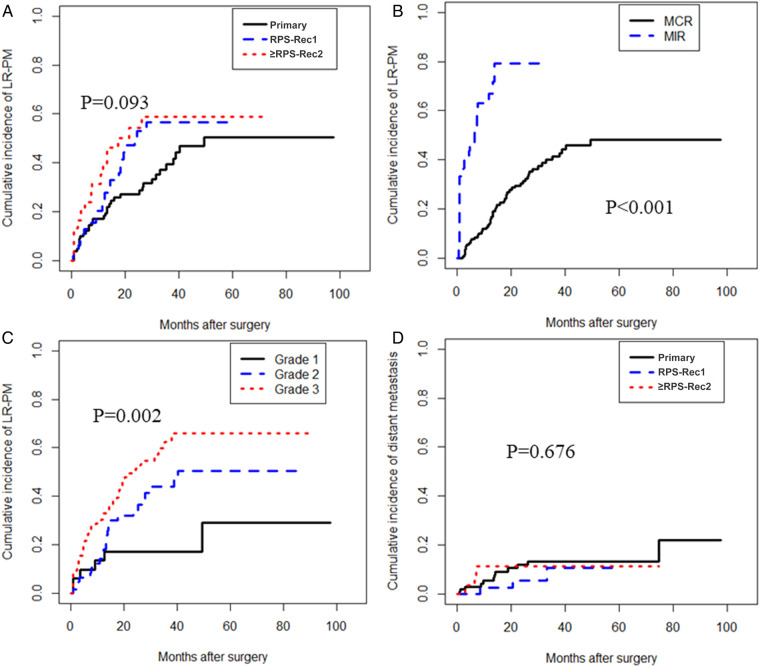
Local recurrence and/or peritoneal metastasis and distant metastasis. (**A**) The cumulative incidence of LR-PM in primary, RPS-Rec1, and ≥RPS-Rec2 patients (primary vs. RPS-Rec1, *P* = 0.164; primary vs. ≥RPS-Rec2, *P* = 0.044). (**B**) The cumulative incidence of LR-PM in MCR and MIR patients (*P* < 0.001). (**C**) The cumulative incidence of LR-PM in different grade RPS patients (grade 1 vs. grade 2, *P* = 0.066; grade 1 vs. grade 3, *P* = 0.001). (**D**) The cumulative incidence of distant metastasis in primary, RPS-Rec1, and ≥RPS-Rec2 patients (primary vs. RPS-Rec1, *P* = 0.388; primary vs. ≥RPS-Rec2, *P* = 0.913). LR-PM, local recurrence and/or peritoneal metastasis; MCR, macroscopically complete resection; MIR, macroscopically incomplete resection.

The cumulative LR-PM rate in the RPS-Rec1 group was not significantly different from that of the primary group, both of which were significantly lower than that of the ≥RPS-Rec2 group (primary vs. RPS-Rec1, *P* = 0.164; primary vs. ≥RPS-Rec2, *P* = 0.044) (Figure 3A). The cumulative 5-year LR-PM rates of the primary, RPS-Rec1, and ≥RPS-Rec2 groups were 50.4% (95% CI: 40.6%–60.1%), 56.5% (95% CI: 47.0%–65.9%), and 58.9% (47.9%–69.9%), respectively (primary vs. RPS-Rec1, *P* = 0.504; primary vs. ≥RPS-Rec2, *P* = 0.869). The multivariate analyses showed that MIR [hazard ratio (HR): 4.30; 95% CI: 2.34–7.89; *P* < 0.001] (Figure 3B) and high-grade RPS (grade 1 vs. grade 2; HR: 1.80; 95% CI: 0.72–4.51; *P* = 0.212; grade 1 vs. grade 3; HR: 3.79; 95% CI: 1.54–9.29; *P* = 0.004) (Figure 3C) were independent risk factors for LR-PM (Table 2).

**Table 2:** Univariate and multivariate analyses of local recurrence and/or peritoneal metastasis (LR-PM), progression-free survival (PFS), and overall survival (OS).

Characteristics	LR-PM			PFS			OS		
	Univariate analysis	Multivariate analysis		Univariate analysis	Multivariate analysis		Univariate analysis	Multivariate analysis	
	*P* value	HR (95% CI)	*P* value	*P* value	HR (95% CI)	*P* value	*P* value	HR (95% CI)	*P* value
Age (continuous variable)	0.027	0.98 (0.96–1.00)	0.096	0.279			0.450		
Sex (male vs. female)	0.310			0.537			0.894		
Presentation									
primary vs. RPS-Rec1	0.127	0.72 (0.36–1.45)	0.358	0.160	0.76 (0.43–1.32)	0.324	0.579	0.72 (0.36–1.43)	0.344
primary vs. ≥RPS-Rec2	0.001	1.16 (0.54–2.52)	0.705	<0.001	1.31 (0.70–2.45)	0.396	0.001	1.33 (0.65–2.73)	0.439
Perioperative therapy (yes vs. no)	0.035	1.01 (0.46–2.24)	0.973	0.012	1.06 (0.55–2.05)	0.870	0.083	1.18 (0.53–2.67)	0.684
Tumor number (single vs. multiple)	<0.001	1.85 (0.93–3.66)	0.079	<0.001	1.56 (0.89–2.73)	0.123	0.007	1.05 (0.52–2.10)	0.897
Tumor size (continuous variable)	0.004	1.01 (0.99–1.03)	0.302	<0.001	1.01 (0.99–1.03)	0.060	<0.001	1.02 (1.01–1.04)	0.010
MCR (yes vs. no)	<0.001	4.30 (2.34–7.89)	<0.001	<0.001	2.86 (1.69–4.84)	<0.001	<0.001	2.20 (1.20–4.06)	0.011
FNCLCC grade									
1 vs. 2	0.047	1.80 (0.72–4.51)	0.212	0.045	1.57 (0.76–3.24)	0.225	0.070	1.90 (0.76–4.75)	0.170
1 vs.3	<0.001	3.79 (1.54–9.29)	0.004	<0.001	3.42 (1.70–6.87)	0.001	0.003	3.38 (1.41–8.11)	0.006

CI, confidence interval; FNCLCC, Federation Nationale des Centres de Lutte Contre le Cancer criteria; HR, Hazard Ratio; MCR, macroscopically complete resection; RPS, retroperitoneal sarcoma; RPS-Rec1, first recurrent RPS; ≥RPS-Rec2, ≥2 recurrences.

There was no significant difference in the cumulative incidence of distant metastasis in the primary, RPS-Rec1, and ≥RPS-Rec2 groups (primary vs. RPS-Rec1, *P* = 0.388; primary vs. ≥RPS-Rec2, *P* = 0.913) (Figure 3D). The cumulative 5-year distant metastasis rates of the primary, RPS-Rec1, and ≥RPS-Rec2 groups were 13.4% (95% CI: 10.5%–16.3%), 10.6% (95% CI: 5.3%–15.9%), and 11.2% (95% CI: 7.0%–15.5%), respectively (primary vs. RPS-Rec1, *P* = 0.607; primary vs. ≥RPS-Rec2, *P* = 0.996).

### PFS and OS

**Figure 4:**
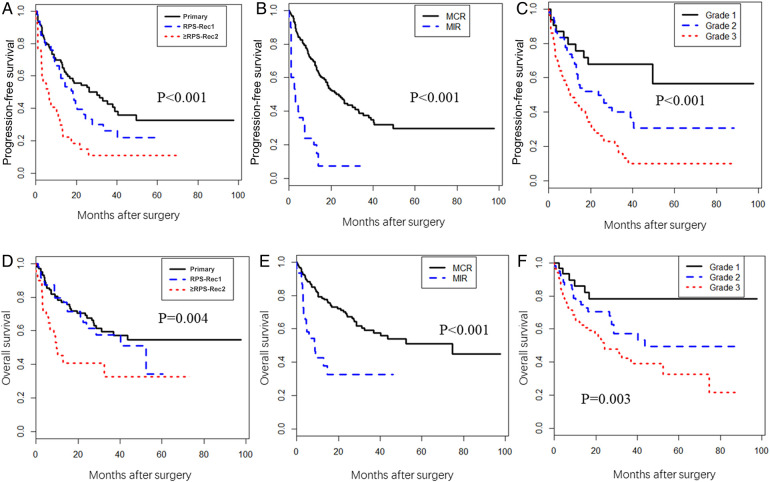
Progression-free survival (PFS) and overall survival (OS). (**A**) The PFS in primary, RPS-Rec1, and ≥RPS-Rec2 patients (primary vs. RPS-Rec1, *P* = 0.151; primary vs. ≥RPS-Rec2, *P* < 0.001). (**B**) The PFS in MCR and MIR patients (*P* < 0.001). (**C**) The PFS in different grade RPS patients (grade 1 vs. grade 2, *P* = 0.042; grade 1 vs. grade 3, *P* < 0.001). (**D**) The OS in primary, RPS-Rec1, and ≥RPS-Rec2 patients (primary vs. RPS-Rec1, *P* = 0.599; primary vs. ≥RPS-Rec2, *P* = 0.001). (**E**) The OS in MCR and MIR patients (*P* < 0.001). (**F**) The OS in different grade RPS patients (grade 1 vs. grade 2, *P* = 0.069; grade 1 vs. grade 3, *P* = 0.002). MCR, macroscopically complete resection; MIR, macroscopically incomplete resection.

The PFS of the RPS-Rec1 group was not significantly different from that of the primary group, both of which were significantly superior to that of the ≥RPS-Rec2 group (primary vs. RPS-Rec1, *P* = 0.151; primary vs. ≥RPS-Rec2, *P* < 0.001) (Figure 4A). The 5-year PFS of the primary, RPS-Rec1, and ≥RPS-Rec2 groups were 32.6% (95% CI: 22.2%–47.8%), 21.9% (95% CI: 11.2%–42.6%), and 11.2% (3.9%–32.3%), respectively (primary vs. RPS-Rec1, *P* = 0.182; primary vs. ≥RPS-Rec2, *P* = 0.021). MIR (HR: 2.86; 95% CI: 1.69–4.84; *P* < 0.001) (Figure 4B) and high-grade RPS (grade 1 vs. grade 2; HR: 1.57; 95% CI: 0.76–3.24; *P* = 0.225; grade 1 vs. grade 3; HR: 3.42; 95% CI: 1.70–6.87; *P* = 0.001) (Figure 4C) were independent risk factors for PFS (Table 2).

The OS of the RPS-Rec1 group was not significantly different from that of the primary group, both of which were significantly superior to that of the ≥RPS-Rec2 group (primary vs. RPS-Rec1, *P* = 0.599; primary vs. ≥RPS-Rec2, *P* = 0.001) (Figure 4D). The 5-year OS of the primary, RPS-Rec1, and ≥RPS-Rec2 groups were 54.5% (95% CI: 43.6%–68.1%), 34.1% (95% CI: 14.1%–82.4%), and 32.6% (17.2%–61.7%), respectively (primary vs. RPS-Rec1, *P* = 0.021; primary vs. ≥RPS-Rec2, *P* = 0.036). MIR (HR: 2.20; 95% CI: 1.20–4.06; *P* = 0.011) (Figure 4E), high-grade RPS (grade 1 vs. grade 2; HR: 1.90; 95% CI: 0.76–4.75; *P* = 0.170; grade 1 vs. grade 3; HR: 3.38; 95% CI: 1.41–8.11; *P* = 0.006) (Figure 4F), and a larger tumor (HR: 1.02; 95% CI: 1.01–1.04; *P* = 0.010) were independent risk factors for OS (Table 2).

## Discussion

The outcomes of patients with primary RPS is significantly better than that of patients with recurrent RPS, and with the subsequent local recurrence, the complete resection rate decreased significantly (3, 12). The treatment of patients with recurrent RPS is a challenging conundrum in sarcoma centers worldwide. However, there are no evidence-based guidelines from any large study to guide clinicians in the treatment of recurrent RPS. We compared patients with RPS-Rec1, primary RPS, and ≥RPS-Rec2, to evaluate the value of *en bloc* resection of tumor and adjacent organs and select cases to receive extended resection in patients with recurrent RPS.

One of the risks of resection of locally recurrent tumors is the associated morbidity, and there is limited data on the postoperative morbidity profile of resection performed for local recurrence (20). In this study, there were no significant differences in the operation time, blood loss volume, incidence of grade III–V POM, reoperation rates, and perioperative mortality between the primary and RPS-Rec1 groups. Although there was no significant difference in the number of resected organs between the primary, RPS-Rec1, and ≥RPS-Rec2 groups, the operation time, blood loss volume, and incidence of grade III–V POM in the ≥RPS-Rec2 group were significantly higher than those in the primary group or the RPS-Rec1 group (Figure 1). This is consistent with the findings of Lehnert et al. (21). Multivariate analyses showed that great vessel resection and pancreatoduodenectomy were independent risk factors for grade III–V POM. This suggests that the safety of *en bloc* resection of tumor and adjacent organs in patients with RPS-Rec1 is similar to that of patients with primary RPS; however, this safety is greatly reduced in patients with ≥RPS-Rec2.

Similar to our results, previous study results have shown that each subsequent recurrence is associated with a significantly lower rate of successful complete resection (1, 3, 12, 22). Unresectability usually occurs due to peritoneal implants or extensive vascular involvement (2). In this study, each subsequent recurrence was associated with a significantly higher rate of multiple tumors. van Dalen et al. reported that a single local recurrence was associated with improved survival following resection of the first local recurrence (23). Conversely, Grobmyer et al. reported that multifocality did not affect OS in a multivariate analysis (12). In our study, the presence of multiple tumors was not an independent risk factor for PFS and OS, consistent with Grobmyer’s conclusions.

Previous studies have demonstrated that RPS can invade adjacent organs (15, 24, 25). Our results show that recurrent RPS invades more adjacent organs and surrounding fat tissue than primary RPS. Recurrent RPS, especially ≥RPS-Rec2, invades the bowel or parenchyma of the solid organs more than surrounding fat tissue (Figure 2). The tendency of invasion of adjacent organs is stronger in recurrent RPS than primary RPS. Therefore, to avoid residual tumor tissues after surgical resection, especially for patients with recurrence, an extended resection involving adjacent organs should be performed. This can help in achieving complete resection and reducing postoperative recurrence.

LR-PM of RPS is the leading cause of death in patients with RPS (3, 8, 9). In this study, the cumulative LR-PM rate of the ≥RPS-Rec2 group was significantly higher than that of the primary group, and there was no significant difference between the RPS-Rec1 and primary groups (Figure 3A). Multivariate analyses showed that MIR and high-grade RPS were independent risk factors for LR-PM. This is consistent with the results of previous studies (20, 21, 26). Therefore, to ensure patient safety, achieving MCR by *en bloc* resection of tumor and adjacent organs helps to reduce LR-PM, especially in patients with RPS-Rec1.

Previous studies have shown that histologic subtypes can significantly affect the recurrence pattern of RPS (9). This study also showed that the postoperative cumulative LR-PM rate of patients with high-grade histologic subtype sarcoma is higher. However, there is no clinical evidence to support a more conservative surgical strategy for low-grade sarcoma. Previous studies have shown that even low-grade sarcomas, such as well-differentiated liposarcoma, can invade adjacent organs (15, 24). In addition, in many patients, a reliable pathological diagnosis cannot be obtained preoperatively, leading to more conservative surgical treatment that increases the chances of residual tumors, thus increasing the postoperative recurrence rate. Therefore, to reduce the possibility of postoperative recurrence, we suggest that *en bloc* resection combined with adjacent organ resection should be performed for different histologic subtypes of sarcomas, while definite evidence is pending.

Although the number of patients who received perioperative treatment significantly increased with each subsequent local recurrence, the OS and PFS of the RPS-Rec1 group were not significantly different from those of the primary group, both of which had significantly superior survival rates compared to those of the ≥RPS-Rec2 group. The results of our study suggest that MIR and high-grade RPS are independent risk factors for PFS and OS, which is consistent with results of previous studies (1, 12). The biological behavior of RPS is an important factor influencing the outcome of patients. For RPS-Rec1 patients with good outcome after surgery, the biological behavior usually be favorable. Surgical margin is another factor influencing the outcome of patients. Therefore, identifying patients who can achieve MCR preoperatively and achieve MCR by *en bloc* resection of tumor and adjacent organs will help to improve outcomes, especially in patients with RPS-Rec1. However, there is no reliable method to assess preoperatively whether the RPS can be removed completely.

Among the 47 patients with RPS-Rec1, the primary surgery of most patients was not performed in our institution. Therefore, it is difficult to maintain consistent surgical quality for patients undergoing primary surgery in various centers, which leads to the surgical treatment of some RPS-Rec1 patients is similar to “salvage operation”, similar to the primary RPS surgery performed for these patients. This may be the possible reason that the prognosis of patients with RPS-Rec1 is similar to that of patients with primary RPS. This is one of the limitations of this study. In view of the small number of cases at present, it is impossible to verify the impact of primary surgical resection on the prognosis of patients with reoperation after first recurrence through data analysis. Although previous studies have shown that the more organs that are resected in the first operation, the worse the prognosis of patients who undergo reoperation after the first recurrence (14). However, this needs more evidence to be verified. We will further explore this issue in the following research. The results of this study cannot fully prove that *en bloc* resection of tumor and adjacent organs is superior to simple tumor resection in RPS-Rec1 patients. The main purpose of this study is by comparing the safety and efficacy of *en bloc* resection of tumor and adjacent organs in primary, RPS-Rec1, and ≥RPS-Rec2 patients, to evaluate the value of *en bloc* resection of tumor and adjacent organs and select cases in recurrent RPS patients. The results of this study just shows that the safety and effectiveness of *en bloc* resection of tumor and adjacent organs in RPS-Rec1 was not significantly different from those in primary RPS but was significantly superior to those in RPS-Rec2, and achieving MCR by *en bloc* resection of tumor and adjacent organs can significantly improve the outcomes. As for whether *en bloc* resection of tumor and adjacent organs is better than simple tumor resection in patients with RPS-Rec1, it needs to be further confirmed by multi center, larger sample size, prospective and more rigorous clinical studies.

Although the risk of surgery in patients with RPS-Rec1 is comparable to that of patients with primary RPS, in theory, it is more difficult to perform surgical resection after recurrence. It is technically challenging due to loss of original planes, distortion of anatomic relationships, and vascular involvement (7). Therefore, although our study shows that the cumulative LR-PM rate, PFS, and OS in patients with RPS-Rec1 are comparable to that in patients with primary RPS after *en bloc* resection of tumor and adjacent organs, this cannot be the basis for conservative surgery in patients with primary RPS. It is still necessary to achieve complete resection by extending the resection to the involved adjacent organs at the time of the first surgery to reduce recurrence in patients with primary RPS.

## Conclusions

The safety and effectiveness of *en bloc* resection of tumor and adjacent organs in RPS-Rec1 was not significantly different from those in primary RPS but was significantly superior to those in RPS-Rec2. Achieving MCR by *en bloc* resection of tumor and adjacent organs can significantly improve the outcomes. Therefore, for patients with RPS-Rec1, *en bloc* resection of tumor and adjacent organs is advisable, and comparable outcomes to patients with primary RPS can be achieved, particularly in those in whom a macroscopically complete resection is achieved.

## Data Availability

The datasets used and/or analyzed during the current study are available from the corresponding author on reasonable request.
