# Supplementary material for: Patients with first recurrent retroperitoneal sarcoma that can be macroscopically completely resected can achieve comparable outcomes with that of primary patients after en bloc resection of tumor and adjacent organs
Source: Front Surg. 2022 Sep 7;9:956384. doi: 10.3389/fsurg.2022.956384 (PMC9489918; doi:10.3389/fsurg.2022.956384)
Supplement: Supplementary file 1 [file Table_1_v1.docx]

| **Reasons for the failure of MCR** | n |
| --- | --- |
| **Primary** | 7 |
| Rupture due to fragile tumor | 2 |
| Cut-through tumors due to difficulty in en bloc resection (large RPS, extending into obturator foramen, surrounding the abdominal aorta and inferior vena cava) | 3 |
| Invasion of ilium | 1 |
| Invasion of sacrum | 1 |
| **RPS-Rec1** | 10 |
| Rupture due to fragile tumor | 3 |
| Invasion of ilium | 2 |
| Multiple implant tumors | 2 |
| Cut-through tumors due to difficulty in en bloc resection (large RPS, surrounding the abdominal aorta and inferior vena cava) | 2 |
| The right kidney is surrounded by RPS and can not be radically resected even if combined with right nephrectomy. | 1 |
| **≥RPS-Rec2** | 13 |
| Cut-through tumors due to difficulty in en bloc resection (large RPS, surrounding the abdominal aorta) | 6 |
| Invasion of spine | 3 |
| Multiple implant tumors | 3 |
| Invasion of choledochojejunostomy at primary operation | 1 |

Supplemental Table 1. Reasons for the failure of macroscopically complete resection.

RPS, retroperitoneal sarcoma; MCR, macroscopically complete resection.
