# Supplementary material for: Patients with first recurrent retroperitoneal sarcoma that can be macroscopically completely resected can achieve comparable outcomes with that of primary patients after en bloc resection of tumor and adjacent organs
Source: Front Surg. 2022 Sep 7;9:956384. doi: 10.3389/fsurg.2022.956384 (PMC9489918; doi:10.3389/fsurg.2022.956384)
Supplement: Supplementary file 2 [file Table_2_v1.docx]

Supplemental Table 2. The details of the organs resected in each group.

| Resected organs |  | Primary（n=101） |  | RPS-Rec1 (n=47) |  | ≥RPS-Rec2 (n=30) |  |
| --- | --- | --- | --- | --- | --- | --- | --- |
| Colon |  | 76 (75.2%) |  | 43 (91.5%) |  | 23 (76.7%) |  |
| Kidney |  | 68 (67.3%) |  | 25 (53.2%) |  | 10 (33.3%) |  |
| Psoas major |  | 38 (37.6%) |  | 20 (42.6%) |  | 13 (43.3%) |  |
| Diaphragm |  | 35 (34.7%) |  | 20 (42.6%) |  | 15 (50.0%) |  |
| Ureter |  | 73 (72.3%) |  | 28 (59.6%) |  | 11 (36.7%) |  |
| Small intestine |  | 32 (31.7%) |  | 15 (31.9%) |  | 22 (73.3%) |  |
| Pancreas |  | 39 (38.6%) |  | 17 (36.2%) |  | 14 (46.7%) |  |
| Adrenal gland |  | 59 (58.4%) |  | 24 (51.1%) |  | 8 (26.7%) |  |
| Abdominal wall |  | 13 (12.9%) |  | 10 (21.3%) |  | 8 (26.7%) |  |
| Gallbladder |  | 25 (24.8%) |  | 9 (19.1%) |  | 9 (30.0%) |  |
| Inferior vena cava |  | 10 (9.9%) |  | 4 (8.5%) |  | 4 (13.3%) |  |
| Iliac artery |  | 16 (15.8%) |  | 5 (10.6%) |  | 6 (20.0%) |  |
| Iliac vein |  | 14 (13.9%) |  | 5 (10.6%) |  | 5 (16.7%) |  |
| Spleen |  | 29 (28.7%) |  | 15 (31.9%) |  | 3 (10.0%) |  |
| Ovary |  | 14 (13.9%) |  | 7 (14.9%) |  | 4 (13.3%) |  |
| Stomach |  | 21 (20.8%) |  | 9 (19.1%) |  | 13 (43.3%) |  |
| Liver |  | 6 (5.9%) |  | 8 (17.0%) |  | 7 (23.3%) |  |
| Ilium |  | 1 (1.0%) |  | 0 |  | 0 |  |
| Bladder |  | 6 (5.9%) |  | 1 (2.1%) |  | 0 |  |
| rib |  | 4 (4.0%) |  | 2 (4.3%) |  | 2 (6.7%) |  |
| Lung |  | 0 |  | 0 |  | 1 (3.3%) |  |
| Portal vein |  | 2 (2.0%) |  | 0 |  | 1 (3.3%) |  |
| Rectum |  | 3 (3.0%) |  | 1 (2.1%) |  | 4 (13.3%) |  |
| Abdominal aorta |  | 1 (1.0%) |  | 0 |  | 0 |  |
| Pericardium |  | 0 |  | 2 (4.3%) |  | 0 |  |
| Uterus |  | 8 (7.9%) |  | 7 (14.9%) |  | 3 (10.0%) |  |
