# Supplementary material for: Patients with first recurrent retroperitoneal sarcoma that can be macroscopically completely resected can achieve comparable outcomes with that of primary patients after en bloc resection of tumor and adjacent organs
Source: Front Surg. 2022 Sep 7;9:956384. doi: 10.3389/fsurg.2022.956384 (PMC9489918; doi:10.3389/fsurg.2022.956384)
Supplement: Supplementary file 3 [file Table_3_v1.docx]

|  | Primary (n=101) | RPS-Rec1 (n=47) | ≥RPS-Rec2 (n=30) |
| --- | --- | --- | --- |
| **Grade III** | **12 (11.9%)** | **9 (19.1%)** | **8 (26.7%)** |
| Grade B POPF | 6 | 2 | 1 |
| Urinary fistula | 1 | 0 | 0 |
| Abdominal infection | 2 | 4 | 5 |
| Postoperative hemorrhage | 2 | 1 | 2 |
| Thrombosis | 1 | 2 | 0 |
| **Grade IV** | **3 (3%)** | **2 (4.3%)** | **1 (3.3%)** |
| Abdominal infection and hemorrhage | 2 | 2 | 1 |
| Renal insufficiency | 1 | 0 | 0 |
| **Grade V** | **2 (2%)** | **2 (4.3%)** | **3 (10%)** |
| **Total** | **17（16.8%）** | **13（27.7%）** | **12 (40%)** |

Supplemental Table 3. Postoperative morbidity data of primary, RPS-Rec1, and ≥RPS-Rec2 patients.

POPF, postoperative pancreatic fistulas.
